# The effects of nuclear factor‐kappa B in pancreatic stellate cells on inflammation and fibrosis of chronic pancreatitis

**DOI:** 10.1111/jcmm.16213

**Published:** 2020-12-30

**Authors:** Nan Wu, Xiao‐Fan Xu, Jia‐Qi Xin, Jian‐Wei Fan, Yuan‐Yuan Wei, Qing‐Xia Peng, Li‐Fang Duan, Wei Wang, Hong Zhang

**Affiliations:** ^1^ Department of Pathophysiology Shaanxi University of Chinese Medicine Xi'an China; ^2^ Medical Experiment Center Shaanxi University of Chinese Medicine Xi'an China; ^3^ Ningxia Medical University Yinchuan China; ^4^ Department of General Surgery & Research Institute of Pancreatic Disease Ruijin Hospital School of Medicine Shanghai Jiao Tong University Shanghai China

**Keywords:** chronic pancreatitis, fibrosis, inflammatory, nuclear factor‐kappa B, pancreatic stellate cells

## Abstract

The activation of pancreatic stellate cells (PSCs) plays a critical role in the progression of pancreatic fibrosis. Nuclear factor‐kappa B **(**NF‐κB) is associated with chronic pancreatitis (CP). Previous evidence indicated that NF‐κB in acinar cells played a double‐edged role upon pancreatic injury, whereas NF‐κB in inflammatory cells promoted the progression of CP. However, the effects of NF‐κB in PSCs have not been studied. In the present study, using two CP models and RNAi strategy of p65 in cultured PSCs, we found that the macrophage infiltration and MCP‐1 expression were increased, and the NF‐κBp65 protein level was elevated. NF‐κBp65 was co‐expressed with PSCs. In vitro, TGF‐β1 induced overexpression of the TGF‐β receptor 1, phosphorylated TGF‐β1–activated kinase 1 (p‐TAK1) and NF‐κB in the PSCs. Moreover, the concentration of MCP‐1 in the supernatant of activated PSCs was elevated. The migration of BMDMs was promoted by the supernatant of activated PSCs. Further knockdown of NF‐κBp65 in PSCs resulted in a decline of BMDM migration, accompanied by a lower production of MCP‐1. These findings indicate that TGF‐β1 can induce the activation of NF‐κB pathway in PSCs by regulating p‐TAK1, and the NF‐κB pathway in PSCs may be a target of chronic inflammation and fibrosis.

## 1 INTRODUCTION

The pathogenesis of chronic pancreatitis (CP), which refers to irreversible structural and functional damage caused by persistent pancreatic inflammation, remains unclear. Previous research demonstrated that activated pancreatic stellate cells (PSCs) play a very important role in the progression of CP.^1^ During the progression of CP, quiescent PSCs are activated by abundant inflammatory mediators including cytokines, such as tumour necrosis factor and transforming growth factors (TGFs) produced by infiltrating inflammatory cells or the PSCs themselves.^2^ Activated PSCs can produce multiple extracellular matrix (ECM), which leads to pancreatic fibrosis and ultimately dysfunction of the exocrine and endocrine glands.^3^ Therefore, the activation of PSCs is considered a critical step in the development of pancreatic fibrosis.^4^, ^5^ Although TGF‐beta 1 (TGF‐β1) is thought to be the most likely regulator of PSC activation and proliferation,^3^ its mechanism in regulating the downstream signalling pathway is unclear. Experiments showed that TGF‐β1 signal transduction was linked to the transcription factor nuclear factor‐kappa B (NF‐κB); however, the results were contradictory, depending on the different cell types.^6^, ^7^ In one study, TGF‐β inhibited the activation of NF‐κB by increasing the expression of inhibitor of κB‐alpha (IκB‐α) in both salivary gland and breast cancer cells.^6^ However, in rat hepatic stellate cells, TGF‐β appeared to induce the activation of NF‐κB.^7^ In PSCs, the influence of TGF‐β on NF‐κB activation remains unclear.

The transcription factor NF‐κB plays a crucial role in regulating inflammation, proliferation and apoptosis.^8^ In a previous study, Steinle et al^9^ reported that the NF‐κBp65 DNA‐binding activity was elevated in a murine model of acute pancreatitis induced by cerulein. Recently, some research studies attempted to explore the relationship between NF‐κB and CP.^10^, ^11^ Matthias et al^11^ indicated that NF‐κBp65 knockout in pancreatic acinar cells promoted fibrosis in a murine model of CP induced by cerulein. At the same time, they found that p65 knockout in macrophages reduced the production of cytokines and alleviated fibrosis in CP. Thus, their results implied that NF‐κB expression in acinar cells provided protection against CP progression, whereas NF‐κB in myeloid cells aggravated the development of CP. In contrast, Huang et al^10^ proposed that sustained activation of NF‐κB in pancreatic acinar cells aggravated the severity of CP. They reported that mice overexpressing IκB kinase 2 (IKK2) in pancreatic acinar cells showed spontaneous acinar damage, obvious inflammation and fibrosis, in addition to NF‐κB overexpression. Furthermore, after the administration of cerulein, the severity of pancreatitis was greater in mice overexpressing IKK2 compared with that observed in wild‐type mice. However, the potential role of NF‐κB in regulating the function of PSCs and inflammatory microenvironment during the progression of CP remains unclear.

In the present study, we used two CP models and a small interfering RNA (siRNA)‐lipotransfection strategy to inhibit p65, a dominant subunit of NF‐κB dimers,^10^ in isolated and cultured PSCs. Our aim was to shed light on the effect and mechanism of NF‐κB in PSCs, and identify a target for inflammation and fibrosis during the progression of CP.

## 2 MATERIAL AND METHODS

### 2.1 Animals

Kunming mice and C57BL/6 mice were purchased from the Experimental Animal Center of Xi'an Jiaotong University (Xi'an, China). All experimental procedures adhered strictly to the animal care and handling guidelines of the Committee on Animal Care of Shaanxi University of Chinese Medicine (Xi'an, China).

### 2.2 Murine models of CP

Two murine models of CP were induced by administration of cerulein (C9026; Sigma‐Aldrich) or L‐arginine (A6969; Sigma‐Aldrich). The C57BL/6 mice were intraperitoneally injected with cerulein (50 μg/kg, six times per day, 3 d/wk) for 6 weeks. The Kunming mice were intraperitoneally injected with 20% l‐arginine (3 g/kg, twice per day, 1 d/wk) for 6 weeks. A control group comprised mice injected with the same amount of sterilized saline. The mice were killed at weeks 2, 4 and 6 after modelling.

### 2.3 Histological analysis

The pancreatic tissues were immediately immersed in 4% paraformaldehyde for 12 hours, dehydrated, embedded in paraffin and sectioned (2 μm). Haematoxylin and eosin and Masson's trichrome staining were performed. Pancreatic sections were assessed at 20× objective magnification over 10 separate fields to determine the severity of pancreatitis by scoring for oedema, inflammation, atrophy, necrosis and fibrosis.

### 2.4 Immunohistochemistry

Immunohistochemistry (IHC) was performed using a ready‐to‐use SABC kit (Boster Biological Technology Co, Ltd). The sections were incubated with a murine monoclonal anti‐p65 antibody (Santa Cruz Biotechnology, Inc), a monoclonal rat anti‐F4/80 antibody (Santa Cruz) and a rabbit MCP‐1 antibody (Biosynthesis Biotechnology Co., Ltd), all for 48 hours at 4°C. The corresponding secondary antibodies (antimouse secondary antibodies for p65 antibody, anti‐rat secondary antibodies for F4/80 antibody, anti‐rabbit secondary antibodies for MCP‐1 antibody) were subsequently incubated for 1 hour at room temperature. Images were captured using a ZEISS Imager A1 Microscope (Carl Zeiss AG).

### 2.5 Immunofluorescence

For immunofluorescence double labelling, the sections were incubated with a mouse monoclonal anti‐α‐SMA antibody (sc‐53015; Santa Cruz, 1:300) and a rabbit anti‐NF‐κB p65 antibody (sc‐109; Santa Cruz, 1:200) or a rabbit anti‐MCP‐1 antibody (bs1101R; Bioss, 1:200) for 48h at 4°C. Antimouse DyLight 594 (BA1141; Boster, 1:500) and anti‐rabbit DyLight 488 (BA1127; Boster, 1:400) fluorescein secondary antibodies were incubated for 1 hour. Finally, the nuclei were counterstained with 4′,6‐diamidino‐2‐phenylindole (DAPI, Roche) for 5 minutes. Images were captured with a laser scanning confocal microscope (Olympus FV3000).

In vitro, PSCs cultured on 20 × 20‐mm slides were fixed in 4% paraformaldehyde, permeabilized in 0.5% Triton X‐100 (Solarbio Life Sciences Co., Ltd), blocked (normal goat serum and 0.5% Triton X‐100) and incubated with a rabbit anti‐p65 antibody or anti‐α‐SMA antibody at 4°C overnight. The cells were incubated for 1 hour with goat anti‐rabbit fluorescein isothiocyanate secondary antibody, and the nuclei were counterstained with DAPI. Images were captured with a ZEISS Imager A2 Fluorescence Microscope (Carl Zeiss AG).

### 2.6 Isolation of PSCs

For the isolation of PSCs, the Kunming mice (n = 6, aged 6‐8 weeks) were killed using diethyl ether in accordance with the standard procedures.^12^ The second passage of PSCs was used in subsequent analyses.

### 2.7 Isolation of bone marrow–derived macrophages (BMDMs)

For the isolation of BMDMs, the Kunming mice (n = 6, aged 6‐8 weeks) were killed and immersed in 75% ethanol. The skin was removed from the lower part of the body, and the remaining pelvic tissue and femoral bone tissue were cleaned, followed by separation at the knee joint. The bones were subsequently immersed in Dulbecco's phosphate‐buffered saline for 5 minutes and placed in RPMI 1640 until the next step. Each end of the bone was then cut off, and the bone marrow from both ends of the bone was rinsed using a 1‐ml syringe filled with RPMI 1640 medium. The bone marrow was collected in a 50‐ml centrifuge tube, and the cells were filtered using a 70‐μm sieve. The red blood cells were removed by red blood cell lysate, and the cells were washed twice with RPMI 1640 medium. Subsequently, 2 × 10^6^ cells were added to 4 mL of complete medium and seeded in 35‐mm culture dishes. After 16 hours of inoculation, adherent cells were discarded, and the cells in the supernatant were collected and re‐seeded in a six‐well plate at 1 × 10^6^, followed by incubation with medium containing 50 ng of macrophage colony‐stimulating factor. Subsequently, 50% of the cell culture medium was replaced with a new 50% cell culture solution containing 50 ng of macrophage colony‐stimulating factor on day 4. BMDMs obtained on day 5 of culture were identified through flow cytometry using macrophage markers (CD11b and F4/80). The cell density was approximately 1 × 10^6^, and the purity of the macrophages was >98%.

### 2.8 Scratch wound assay

The BMDMs were seeded in a six‐well plate with serum‐free culture medium at 1 × 10^6^. Cell proliferation was completely inhibited by the administration of 10 µg/mL of mitomycin C (Invitrogen) for 1 hour. A straight scratch was performed using the tip of a P200 pipette. The cells were subsequently washed thrice with phosphate‐buffered saline and cultured in different culture supernatants, including a control group, TGF‐β1 without PSC group, TGF‐β1–stimulated PSC group, control RNA interference (RNAi) plus TGF‐β1 group, and NF‐κBp65 RNAi plus TGF‐β1 group. After incubation for 24 hours, the gap width of the scratch repopulation was measured and compared with the initial gap size (0 hour).

### 2.9 RNAi strategy

The effectiveness of Lipofectamine 3000 (Thermo Fisher Scientific) in transfecting siRNA into PSCs was examined using fluorescein amidite fluorescein‐labelled negative control siRNA. Three sequences of NF‐κBp65 siRNA were pre‐transfected for screening. NF‐κBp65 siRNA (final dilution: 60 pmol/mL) and Lipofectamine 3000 were dissolved (1:1 ratio) in Opti‐MEM (Gibco). The suppression of p65 mRNA efficiency was detected using the real‐time PCR. The most effective p65 siRNA (p65 1663) was selected for subsequent experiments. The sequences of the siRNAs are shown in Table 1.

**Table 1:** siRNA sequences used for RNA interference

siRNA	Sense	Antisense
p65‐435	5′‐GCAUGCGAUUCCGCUAUAATT‐3′	5′‐UUAUAGCGGAAUCGCAUGCTT‐3′
p65‐1663	5′‐CCUGCAGUUUGAUGCUGAUTT‐3′	5′‐AUCAGCAUCAAACUGCAGGTT‐3′
p65‐1882	5′‐GGAGUACCCUGAAGCU‐AUATT‐3′	5′‐ UAUAGCUUCAGGGUACUCCTT‐3′
N.A Ctrl	5′‐UAUAGCUUCAGGGUACUCCTT‐3′	5′‐ACGUGACACGUUCGGAGAATT‐3′
N.A FAM	5′‐UAUAGCUUCAGGGUACUCCTT‐3′	5′‐ACGUGACACGUUCGGAGAATT‐3′

### 2.10 Western blot analysis

For Western blotting, lysates from pancreatic tissue (loading protein = 40 µg) or PSCs (loading protein = 20 µg) were separated through sodium dodecyl sulphate‐polyacrylamide gel electrophoresis and transferred to a polyvinylidene difluoride membrane. The membrane was incubated with the respective primary antibody overnight at 4°C and then combined with secondary antibodies conjugated to horseradish peroxidase (BA1054 and BA1050; Boster Biological Technology Co., Ltd.). The primary antibodies used were anti‐p65 (ab131485; Abcam Inc), anti‐phosphorylated p65 (ab131109; Abcam Inc), anti‐α‐SMA (bs10196R; Beijing Biosynthesis Biotechnology Co., Ltd), anti‐IκB‐α (D120138; Sangon Biotech Co., Ltd) or anti‐β‐actin (BM0627; Abcam Inc). Detection and image capturing were performed using an enhanced chemiluminescence illuminating system.

### 2.11 Quantitative real‐time PCR

Total RNA was extracted from pancreatic tissue or cultured PSCs and reverse transcribed using a reverse transcription kit (Takara Biotechnology). The real‐time PCR was performed using a SYBR Green Kit (Takara Biotechnology). Amplification and detection were accomplished using an ABI 7500 fast real‐time PCR system. GAPDH was used as an internal RNA loading control. The primer sequences are shown in Table 2.

**Table 2:** Primer sequences used for real‐time PCR

Gene	Primer forward	Primer reverse
MCP‐1	5′‐GTTGGCTCAGCCAGATGCA‐3′	5′‐AGCGTACTCATTGGGATCATTTG‐3′
NF‐κB p65	5′‐ACTGCCGGGATGGCTACTAT‐3′	5′‐TCTGGATTCGCTGGCTAATGG‐3′
MMP‐1	5′‐GTGAATGGCAAGGAGATGATGG‐3′	5′‐ACGAGGATTGTTGTGAGTAATGG‐3′
TIMP‐1	5′‐CATCTCTGGCATCTGGCATCC‐3′	5′‐CGCTGGTATAAGGTGGTCTCG‐3′
GAPDH	5′‐TGAACGGGAAGCTCACTGG‐3′	5′‐TCCACCACCCTGTTGCTGTA‐3′

### 2.12 Enzyme‐linked immunosorbent assay

The supernatant from the cultured PSCs was measured using enzyme‐linked immunosorbent assay kits (Boster Biological Technology Co., Ltd.) for quantification of the concentration of MCP‐1, without dilution.

### 2.13 Statistical analysis

Data were expressed as the mean ± standard deviation. Statistical analyses were performed using a *t* test and one‐way analysis of variance (ANOVA). A *P* value <.05 was considered statistically significant.

## 3 RESULTS

### 3.1 Pathological changes and overexpression of NF‐κΒ in two different murine models of CP

**Figure 1:**
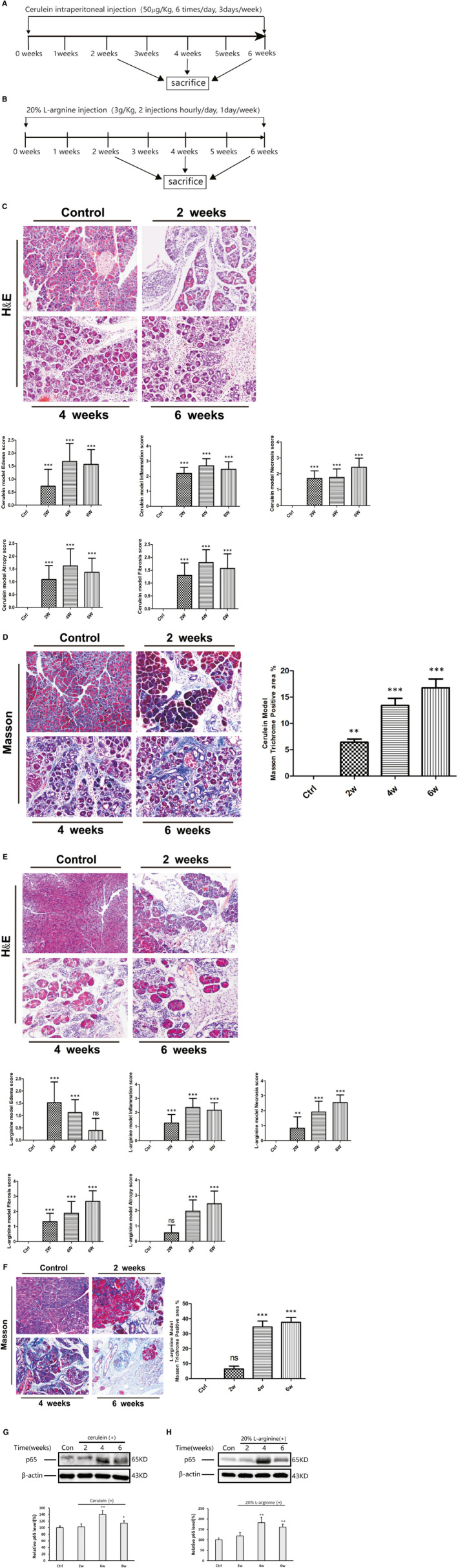
Pathological changes and NF‐κΒ expression in two murine models of CP. A, B, Schematic model for CP. C–F, H&E and Masson's trichrome staining in the pancreas of the CP models induced by cerulein (C, D) or l‐arginine (E, F) at weeks 2, 4 and 6 (original magnification: 200×). Pancreatic pathological score involving oedema, inflammation, atrophy, necrosis and fibrosis (n = 10). The CP group vs the control group: ^**^ *P* < .01, ^***^ *P* < .001. G, H, Western blotting of NF‐κBp65 expression in both murine CP models (representative blot, n = 3). The relative protein abundance was quantified through densitometry analysis. The CP group vs the control group: ^*^ *P* < .05, ^**^ *P* < .01

As shown in Figure 1A,B, the mice were killed at weeks 2, 4 and 6 after receiving an injection of cerulein or l‐arginine. Both murine models showed characteristics of CP, including pancreatic oedema, necrosis, inflammatory cell infiltration, acinar atrophy and fibrosis, as observed by haematoxylin and eosin and Masson's trichrome staining (Figure 1C–F). Pancreatic injury was evaluated based on histopathological changes. Compared with the controls, mice in the CP groups showed typical histological changes in the pancreas with increased histopathological scores. The expression level of NF‐κBp65 protein in pancreatic lysates increased with the development of experimental CP (Figure 1G,H).

### 3.2 Overexpression of NF‐κB detected in activated PSCs in both CP models

**Figure 2:**
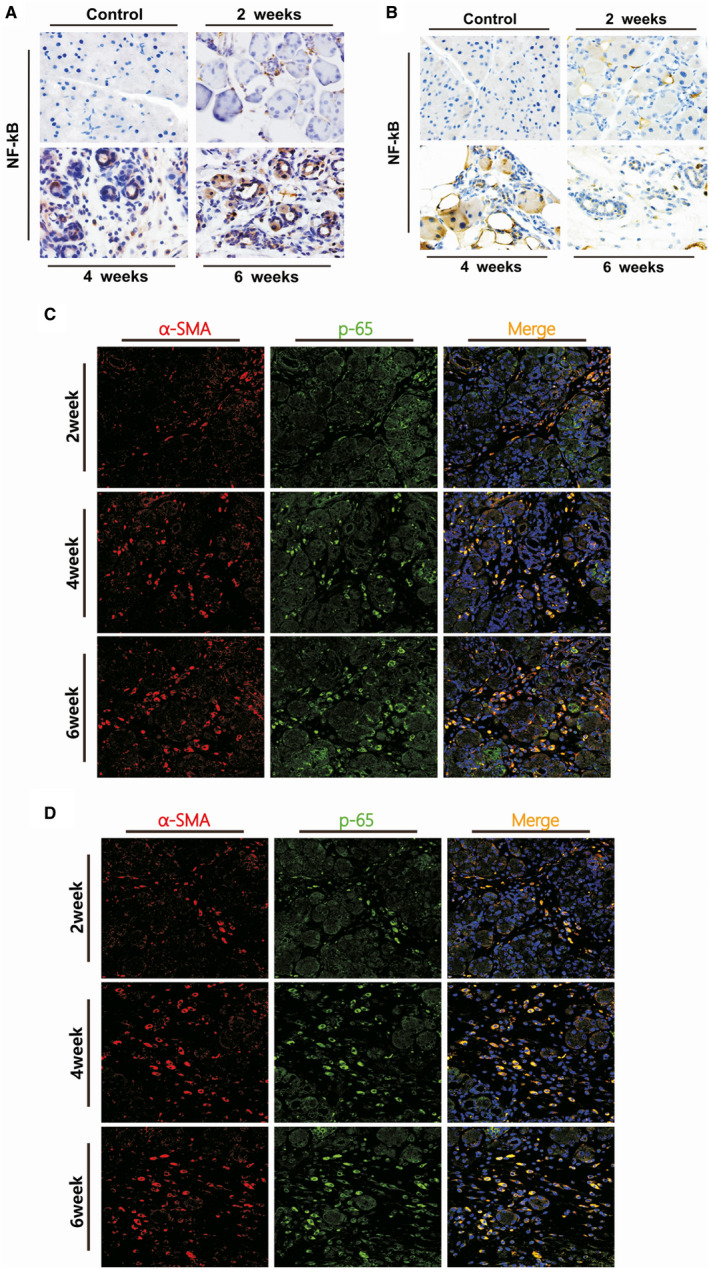
Localization of NF‐κB p65 in the PSCs after the induction of CP at weeks 2, 4 and 6 in both murine models. A, B, IHC staining for NF‐κBp65, the brown colour denoting positive cells (original magnification: 200×). C, D, Immunofluorescence double staining of α‐SMA and p65 in the murine models of CP. DyLight 594–conjugated α‐SMA (red), DyLight 488–conjugated p65 (green), DAPI (blue) and co‐expression areas (orange). Original magnification: 400×

Immunohistochemistry was performed to detect the localization of NF‐κB overexpression in the pancreas. Positive staining of NF‐κBp65 was observed in the acinar to ductal metaplasia lesions and surrounding cells. Apart from previously published report showing p65 expression in acinar cells and infiltrating macrophages^10^, ^11^ (Figure S1), we have observed that p65 was also expressed in fibroblast‐like cells in the CP groups, but not in the control group. The results of immunofluorescence double staining revealed that the co‐staining of p65 and α‐SMA significantly increased in the pancreas of both murine CP models (Figure 2C,D). These findings demonstrate that overexpression of NF‐κB is involved in PSC activation and CP progression.

### 3.3 Overactivation of NF‐κB in isolated PSCs stimulated with TGF‐β1 in vitro

**Figure 3:**
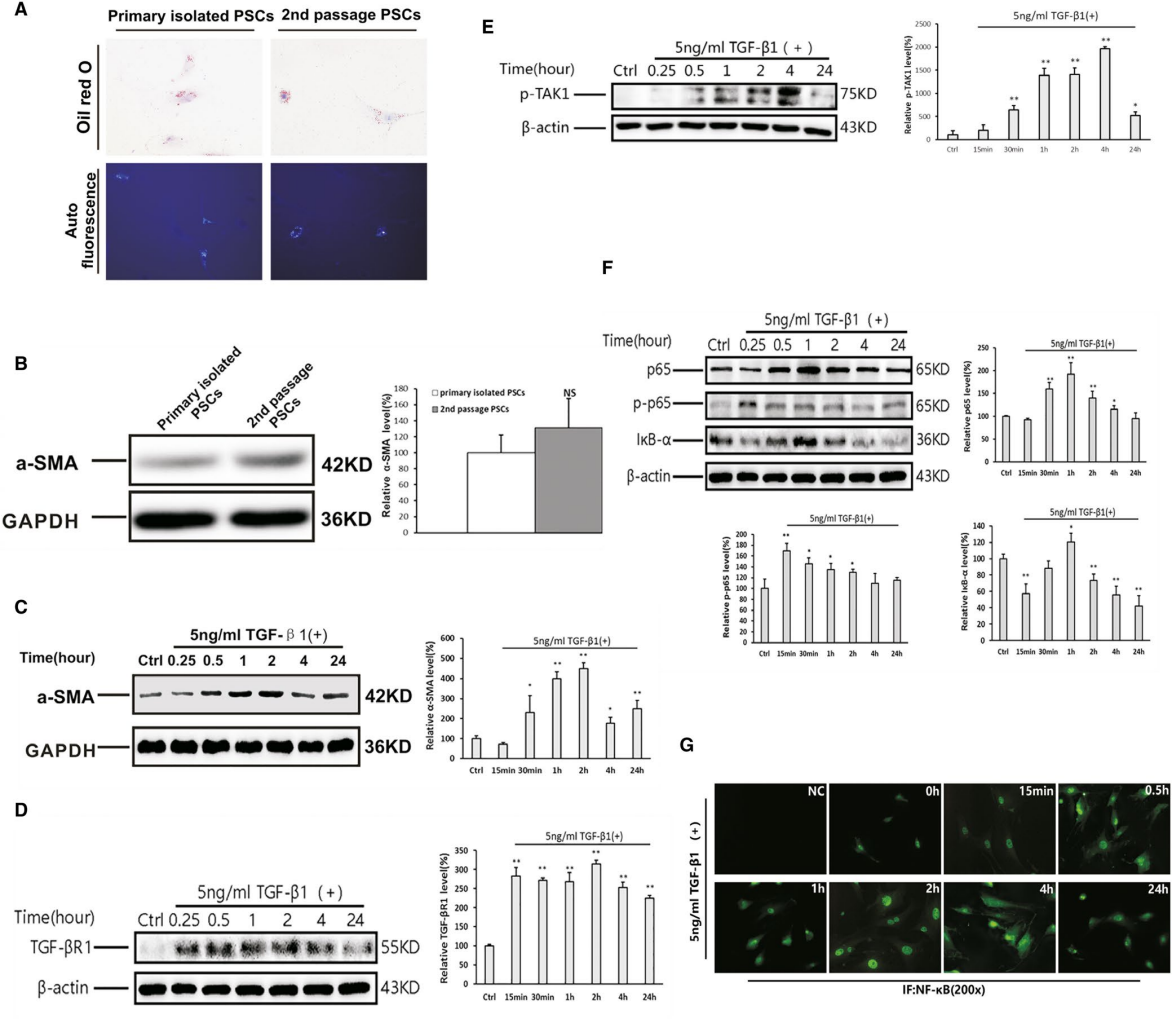
Overactivation of the NF‐κB signalling pathway in the PSCs following stimulation with TGF‐β1. A, Oil Red O staining and autofluorescence in primary isolated and the second passage of PSCs. B, Western blotting analysis of the expression of α‐SMA in primary isolated and the second passage of PSCs at 5 days of culture, with GAPDH used as a loading control. C–F, Western blotting analysis of the expression of α‐SMA (C), TGF‐βR1 (D), p‐TAK1 (E), p65, p‐p65 and IκB‐α (F) in PSCs stimulated with 5 ng/mL of TGF‐β1, with GAPDH or β‐actin used as a loading control (representative blot, n = 3). The relative protein abundance was quantified through densitometry analysis and normalized against GAPDH or β‐actin. The TGF‐β1 treatment group vs the control group: ^*^ *P* < .05, ^**^ *P* < .01. G, Immunofluorescent staining of NF‐κB p65 in the PSCs stimulated with 5 ng/mL of TGF‐β1 at different measurement time‐points. NC means negative control without primary antibody of NF‐κB p65 (original magnification: 200×)

The second passage of PSCs was used in the experiment to obtain a sufficient amount of PSCs for the proteolytic samples. Experiments were performed to identify potential differences between the primary isolated PSCs and the second passage of PSCs at 5 days of culture. We found that both types of cells showed positive staining of Oil Red O and autofluorescence (Figure 3A). The expression of α‐SMA in the second passage of PSCs at 5 days of culture was only slightly higher than that observed in the primary isolated PSCs, without significant difference (Figure 3B). These findings indicate that the activity of the second passage PSCs is similar to that of primary isolated PSCs, and the second passage of PSCs was suitable for subsequent experiments. TGF‐β1 is a potent driver of pancreatic fibrosis; therefore, the PSCs were incubated with 5 ng/mL of TGF‐β1 for 15 minutes, 30 minutes, 1, 2, 4 and 24 hours. The results showed that the expression of α‐SMA was highly elevated after 30 minutes of stimulation with TGF‐β1 and sustained at a higher level for 24 hours, suggesting that TGF‐β1 may augment the activity of PSCs (Figure 3C). The overexpression of TGF‐β receptor 1 in the PSC lysate was observed from 15 minutes of incubation (Figure 3D). At the same time, the expression of p‐TAK1, which is the upstream gene of the NF‐κB signalling pathway, was highly elevated after stimulation with TGF‐β1 from 30 minutes to 4 hours (Figure 3E). The expression of p65 was elevated 30 minutes after stimulation with TGF‐β1, reached a peak after 1 hour and remained at a high level until 4 hours (Figure 3F). The rapid decline in the expression of IκB‐α after stimulation with TGF‐β1 was accompanied by up‐regulation of phosphorylated p65. Similar results were detected in the immunofluorescence staining of NF‐κB p65. As shown in Figure 3G, in the absence of TGF‐β1, NF‐κB p65 was weakly expressed in the cytoplasm of the PSCs. After incubation with TGF‐β1 for 30 minutes‐4 hours, strong staining of NF‐κB p65 was detected in the cytoplasm and nuclei of the PSCs. At 24 hours, the expression of NF‐κB p65 returned to a low level.

### 3.4 Successful knockdown of NF‐κBp65 expression in PSCs through siRNA lipotransfection

**Figure 4:**
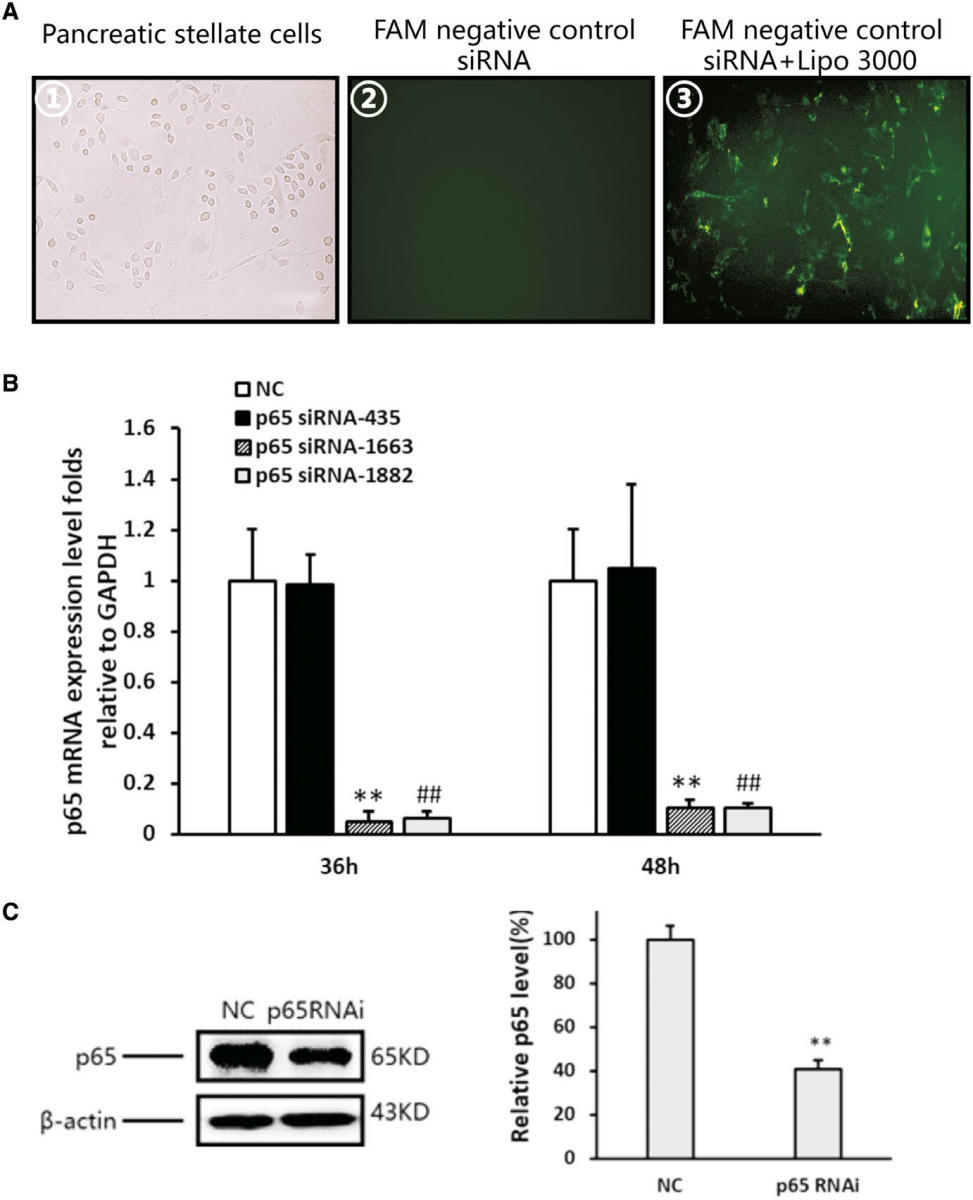
Lipotransfection of NF‐κBp65 siRNA and transfection efficiency. A, ① No transfected PSCs under light microscopy; ② PSCs cultured with FAM‐negative control siRNA without Lipofectamine 3000; and ③ PSCs transfected with FAM‐negative control siRNA using Lipofectamine 3000 (original magnification: 200×). B, Three siRNA sequences of NF‐κBp65 transfection induced alterations in the expression of p65 mRNA in the PSCs. Data shown are percentages compared with that of the negative control siRNA‐transfected cells. p65‐1663 vs the control: ^**^ *P* < .01, p65‐1882 vs the control: ^##^ *P* < .01. C, Western blotting and densitometry analysis showing a reduction in the expression of p65 protein after transfection of p65 siRNA. β‐actin was used as a loading control. p65 siRNA vs the negative control: ^**^ *P* < .01

As shown in Figure 4A, the fluorescein amidite fluorescence signal was detected in the PSCs after 36 hours of transfection with negative control siRNA plus Lipofectamine 3000 (Thermo Fisher Scientific), suggesting that Lipo3000 was effective in transfecting siRNA into these cells. Three siRNA sequences of NF‐κB p65 were transfected into the PSCs to determine their efficiency. The ability of the different siRNAs to inhibit the expression of p65 in PSCs was detected at 36 hours and 48 hours using the real‐time PCR. The primary inhibition was observed using p65 siRNA (p65‐1663), with the most effective inhibition observed at 36 hours (>90% inhibition; Figure 4B). As shown in Figure 4C, the level of p65 protein was markedly inhibited in the si‐p65 (p65‐1663) group compared with that measured in the negative control group. These results suggest that transfection of p65 siRNA can be used to knock down the expression of NF‐κB in PSCs.

### 3.5 Effect of NF‐κBp65 knockdown on PSC activation and fibrosis‐related factors

**Figure 5:**
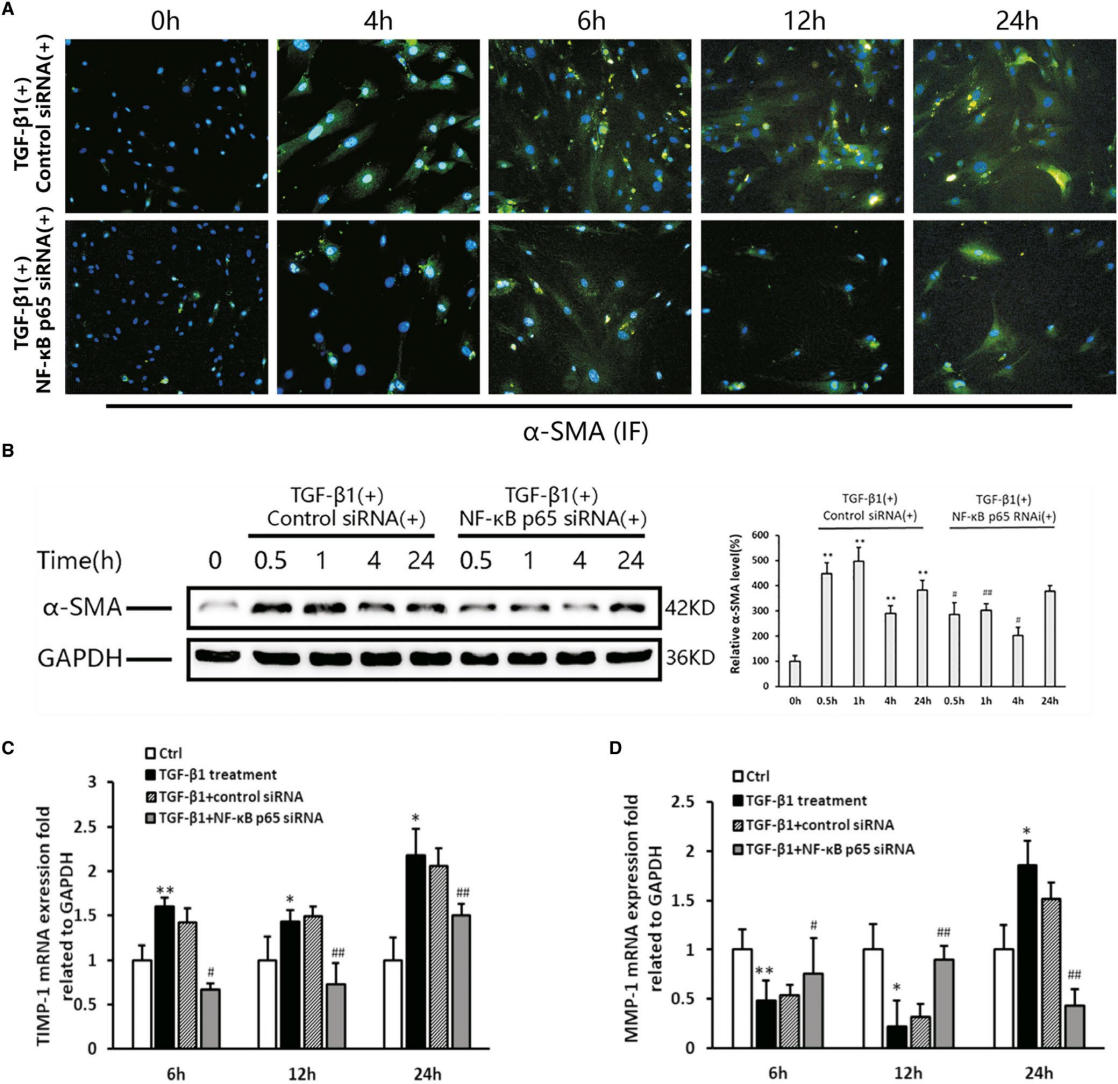
PSC activation and fibrosis‐related factor expression in response to p65 RNAi. A, Immunofluorescent staining of α‐SMA (original magnification: 200×). B, Western blotting and densitometry showing the expression of α‐SMA. GAPDH is shown as a loading control (representative blot, n = 3). The relative protein abundance was quantified through densitometry analysis normalized against GAPDH. The TGF‐β1 group vs the control group: ^*^ *P* < .05, ^**^ *P* < .01; the p65RNAi group vs the control RNAi group: ^#^ *P* < .05, ^##^ *P* < .01. C, D, The MMP‐1 and TIMP‐1 mRNA expression levels in the PSCs were tested using the real‐time PCR (n = 3). The TGF‐β1 group vs the control group: ^*^ *P* < .05, ^**^ *P* < .01; the p65RNAi group or control RNAi group vs the TGF‐β1 treatment group: ^#^ *P* < .05, ^##^ *P* < .01

Activated PSCs were determined by detecting the level of α‐SMA expression via immunofluorescence staining and Western blotting. The results showed that the number of positively stained cells and the level of α‐SMA protein were decreased after NF‐κBp65 knockdown compared with that reported in the control siRNA group (Figure 5A,B). As shown in Figure 5C, the level of matrix metalloproteinase‐1 (MMP‐1) mRNA in the PSCs treated with TGF‐β1 declined at 6 hours (*P* < .01) and 12 hours (*P* < .05), but increased obviously at 24 hours (*P* < .05) compared with that measured in the control group. The knockdown of NF‐κBp65 induced the up‐regulation of MMP‐1 at 6 hours (*P* < .05) and 12 hours (*P* < .01) compared with that observed in the TGF‐β1 group; however, it significantly declined at 24 hours (*P* < .01). The levels of tissue inhibitor of MMP1 (TIMP‐1) in the TGF‐β1 group were elevated at all measurement time‐points. After successful transfection of NF‐κBp65 siRNA, the expression level of TIMP‐1 was reduced compared with that detected in the TGF‐β1 group (Figure 5D).

### 3.6 Effect of NF‐κBp65 knockdown on the production of chemokine MCP‐1 and its potential role in regulating pancreatic inflammation

**Figure 6:**
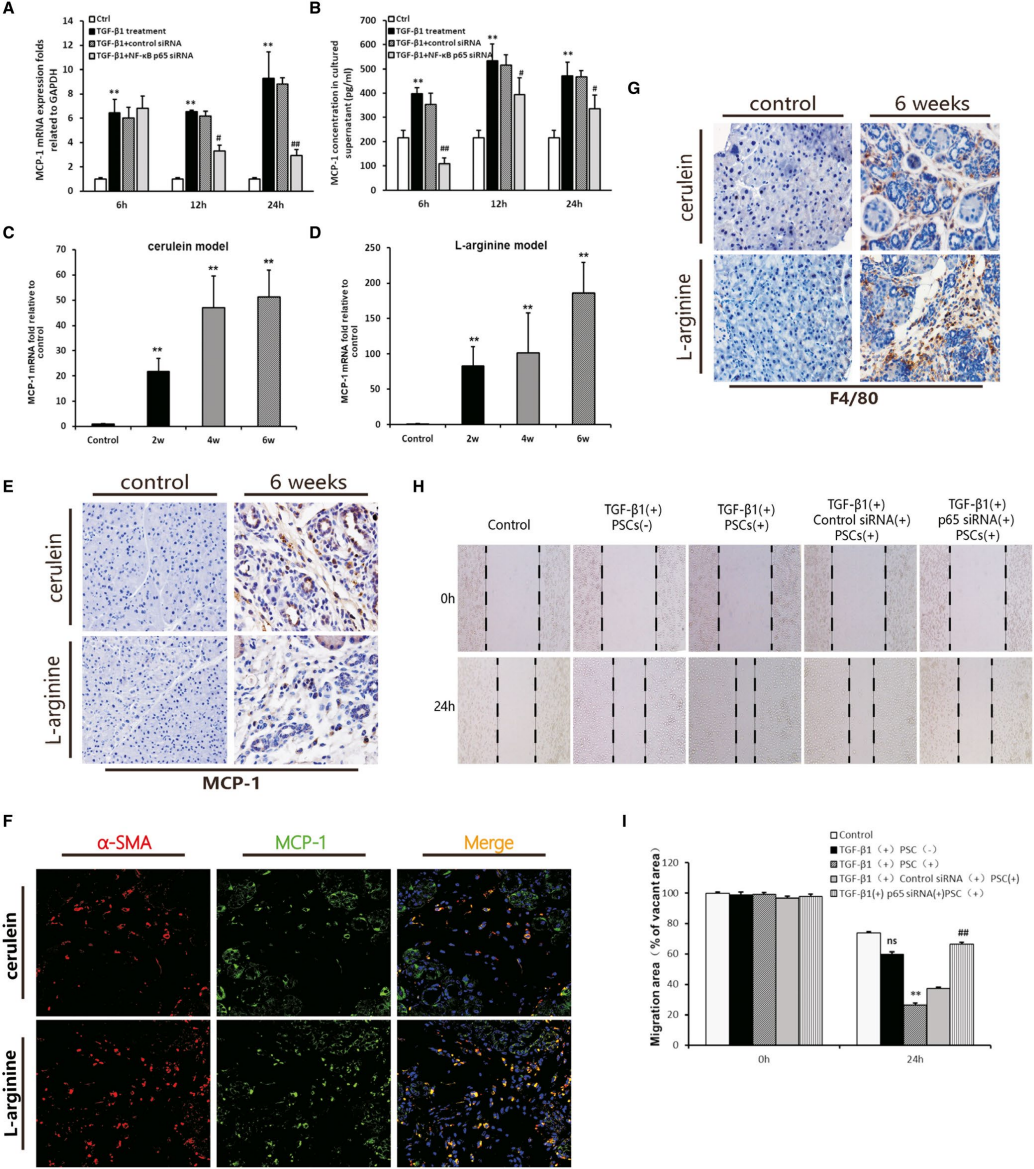
The effects of NF‐κBp65 knockdown in PSC on the production of MCP‐1 and migration of macrophages. A, MCP‐1 mRNA expression levels in the PSCs (n = 3). B, MCP‐1 concentrations in the PSC culture supernatant (n = 3). The TGF‐β1 group vs the control group: *P < .05, **P < .01; the p65RNAi group or control RNAi group vs the TGF‐β1 treatment group: ^#^ *P* < .05, ^##^ *P* < .01. C, D, MCP‐1 mRNA expression levels in the two CP models at weeks 2, 4 and 6 (n = 4). Different time‐points vs the control group: ^**^ *P* < .01. E, IHC staining for MCP‐1 in the two CP models after 6 weeks (original magnification: 200×). F, Immunofluorescence double staining of α‐SMA and MCP‐1 in the murine models of CP. DyLight 594–conjugated α‐SMA (red), DyLight 488–conjugated p65 (green), DAPI (blue) and co‐expression areas (orange). Original magnification: 400×. G, IHC staining for F4/80 (original magnification: 200×). H, Representative images of the scratch wound assay on the migration of BMDMs cultured with different conditional medium of PSC. The black dotted lines indicate the wound borders at the beginning of the assay and those recorded 0 h and 24 h post‐scratching (original magnification: 100×). I, Quantitative analysis of the migration of BMDMs. The relative scratch gap was calculated as the ratio of the remaining scratch gap at 24 h post‐scratching and over that at 0 h (n = 3). The TGF‐β1 with PSC group or TGF‐β1 without PSC group vs the control group: ns (no significance), ^**^ *P* < .01; the p65RNAi group or control RNAi group vs the TGF‐β1 with PSC group: ^##^ *P* < .01

The level of MCP‐1 mRNA expression in PSCs stimulated with TGF‐β1 was increased at 12 hours and 24 hours (*P* < .01 compared with the TGF‐β1–untreated group); however, it was obviously reduced by pre‐treatment with p65 siRNA (*P* < .01). The results of the enzyme‐linked immunosorbent assay showed that the level of MCP‐1 in the supernatant of PSCs stimulated with TGF‐β1 was raised at 12 hours and 24 hours compared with that measured in the control group (*P* < .05). The high level of MCP‐1 in the supernatant of the PSCs was markedly inhibited by pre‐treatment with p65 siRNA (Figure 6A,B). These results suggest that NF‐κBp65 regulated the production of MCP‐1 in PSCs.

The in vivo experiment showed that a high level of MCP‐1 mRNA was observed in the pancreas of the two different CP murine models (Figure 6C,D). The IHC result showed that PSC was one of the MCP‐1‐positively stained cells, in addition to atrophied acinar cells and inflammatory cells (Figure 6E). The immunofluorescence double‐staining result showed that MCP‐1 and a‐SMA co‐expression in the both CP model at 6 weeks (Figure 6F). This result further clarified the expression of MCP‐1 in activated PSC. During the progression of CP, extensive infiltration of inflammatory cells was observed in the pancreas (Figure 1C,E). Further analysis of the macrophage‐specific marker F4/80 demonstrated that infiltration of macrophages increased in both murine models of CP (Figure 6G). However, the relationship and mechanism between the high level of MCP‐1 and macrophage infiltration in the pancreatic microenvironment remain unclear.

**Figure 7:**
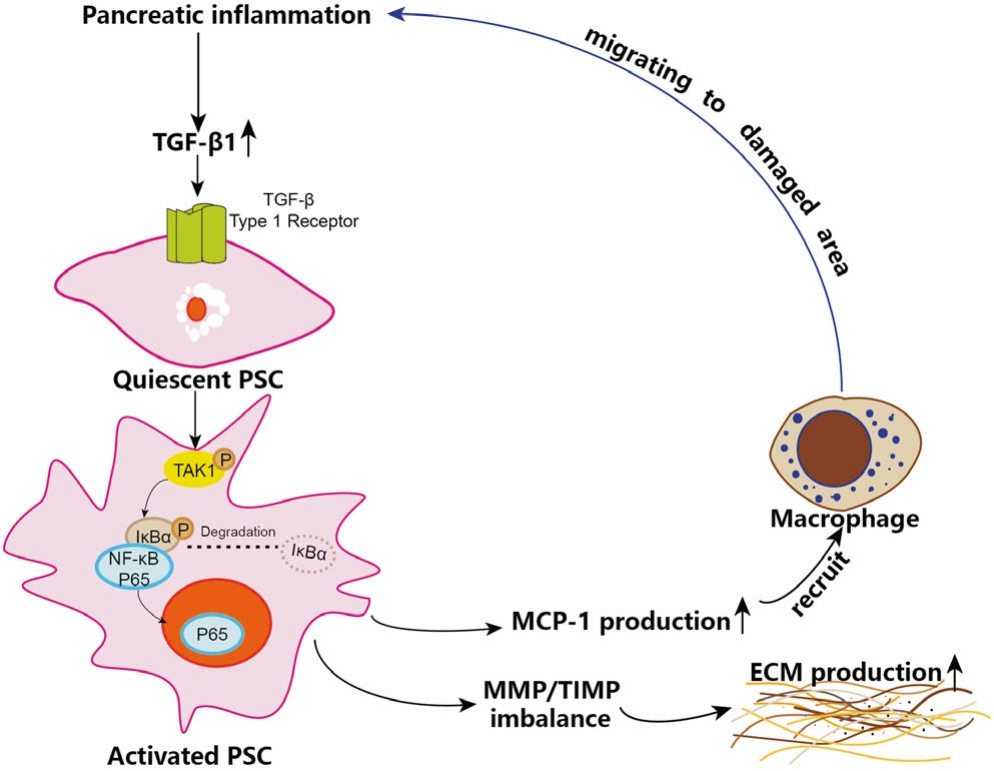
The mechanism of the NF‐κB signalling pathway in PSCs influencing pancreatic inflammation and fibrosis of CP

Given the high level of MCP‐1 detected in the supernatant of the PSCs stimulated by TGF‐β1, as shown in the in vitro experiment, the BMDMs were incubated with the cell culture supernatant of the PSCs stimulated with TGF‐β1 for 24 hours. Additionally, scratch wound healing assays were performed to observe the effect of the PSC culture supernatant on the migration of BMDMs. Compared with the control group, the supernatant from the TGF‐β1–treated PSCs markedly enhanced the migration of BMDMs after treatment for 24 hours (Figure 6H,I). The medium containing an equal concentration of TGF‐β1 (5 ng/mL) without PSCs was collected for the stimulation of BMDMs (24 hours) to rule out the influence of TGF‐β1 itself on the migration of BMDMs. The results showed that the medium with TGF‐β1 alone was unable to significantly accelerate the migration of the BMDMs. These findings suggest that some factors in supernatant, including MCP‐1 produced by activated PSCs, played an important role in the migration of macrophages. Meanwhile, the supernatant of the NF‐κB p65 RNAi group significantly inhibited the migration of BMDMs (Figure 7). The results indicate that migration of macrophages induced by the supernatant of activated PSCs was dependent on the activity of the NF‐κB pathway.

## 4 DISCUSSION

Progressive fibrosis is considered the final pathological manifestation of CP, and PSC activation is an early cellular event in the initiation of pancreatic fibrosis.^12^, ^13^ Previous studies proposed that a variety of factors may stimulate the activation of PSC in a paracrine manner.^14^, ^15^ Others showed that PSCs also possess the ability to secrete cytokines, which accelerated the activation of PSC in an autocrine manner.^16^ Thus, paracrine and autocrine mechanisms work in unison to promote the activation of PSCs, leading to excessive deposition of ECM and pancreatic fibrosis. Previous research demonstrated that TGF‐β1 was one of the strongest pro‐fibrotic cytokines and regulated the activation and proliferation of PSCs in an autocrine or paracrine manner.^13^, ^14^, ^15^ However, the mechanism of TGF‐β1 regulating the activation of PSC and secretion of cytokines remained unclear.

NF‐κB is a crucial transcription factor involved in regulating inflammation, proliferation and apoptosis, whereas the NF‐κB pathway is one of the most important pathways involved in the pathogenesis of CP.^8^ Recently, several studies investigated the role of NF‐κB in different cell types in the progression of CP.^10^, ^11^, ^17^, ^18^ These studies identified several types of cells in the pancreatic inflammatory microenvironment, mainly including acinar cells, inflammatory cells and PSCs. Mattias et al^11^ reported that NF‐κB activation in myeloid cells promoted inflammation and fibrosis in experimental CP. However, NF‐κB in acinar cells provided protection against the progression of CP. In contrast, other studies reported that NF‐κB in acinar cells aggravated the severity of CP.^10^, ^17^, ^18^ These contradictory results indicate that NF‐κB plays diverse roles in the progression of CP and its role depended on the type of cell. Nonetheless, the role of NF‐κB in PSCs has not been investigated. In the present study, we induced two murine models of CP, with similar morphology to that observed in human cases of CP (Figure 1C–F). Overexpression of NF‐κB was observed in both CP models (Figure 1G,H). The IHC results revealed overexpression of NF‐κB in acinar cells, infiltrating macrophages and activated PSCs (Figure 2A,B). The immunofluorescence double‐labelled staining for the co‐expression of NF‐κB p65 and α‐SMA further confirmed that NF‐κB was overexpressed in activated PSCs during the progression of CP (Figure 2C,D).

To investigate the role of NF‐κB specifically in PSCs, we isolated PSCs from healthy mice, cultured and stimulated the second passage of PSCs with TGF‐β1. The results showed that activated PSCs expressed a high level of α‐SMA in response to TGF‐β1 (Figure 3C). At the same time, IκB‐α was abundantly degraded, and p65 phosphorylation was increased. Thereafter, the expression of p65 protein was upregulated and reached a peak at 1 hour after incubation with TGF‐β1. These results suggested that the NF‐κB pathway in PSCs was activated by TGF‐β1 (Figure 3F). However, the mechanism through which TGF‐β1 induced the activation of the NF‐κB pathway in PSCs remains unclear.

In addition, we found that the TGF‐β receptor 1 and p‐TAK1 were rapidly overexpressed after stimulation with TGF‐β1 (Figure 3D,E). In experiments involving other types of cells, TGF‐β1 activated TAK, with subsequent activation of the IKK complex, followed by degradation of phosphorylated IκB‐α and nuclear translocation of NF‐κB.^19^, ^20^ Arsura et al^21^ provided further evidence that TAK1 may participate in TGF‐β–induced activation of NF‐κB. In our experiment, the expression of p‐TAK1 was highly elevated and induced earlier than the expression of NF‐κB after stimulation with TGF‐β1. These findings indicate that the process of NF‐κB activation in PSCs induced by TGF‐β1 may be related to TAK1 phosphorylation.

As previously reported, in the NF‐κB ‘classical pathway’, p65/p50 is the predominant form of NF‐κB heterodimers, and p65 contains a Rel homology domain responsible for DNA binding.^22^ Therefore, in the present study, p65 was selected as a target to inhibit the activity of NF‐κB.^23^ The cells were transfected with p65 siRNA using Lipofectamine 3000 (Thermo Fisher Scientific) to ascertain the role of NF‐κB in the activation of PSC induced by TGF‐β1. After successful transfection of p65 siRNA, PSC activation was significantly decreased, as shown by a reduction in the level of α‐SMA (Figure 5). This finding further indicated that NF‐κB plays an important role in the regulation of PSC activation. Furthermore, shortly after transfection of p65 siRNA, the suppression of MMP‐1 caused by TGF‐β1 was relieved, and the elevation of TIMP‐1 was inhibited (Figure 5). These results suggest that NF‐κB activation in PSCs is responsible for the imbalance between the expression of MMP‐1 and TIMP‐1, both of which are related to the deposition and degradation of ECM in the progression of CP.^24^, ^25^, ^26^

In this study, we found that infiltration of inflammatory cells increased with the progression of pancreatic fibrosis in both CP models (Figure 6G). Many previous studies revealed that the chemokine MCP‐1 contributed to inflammation by recruiting inflammatory cells to injured areas.^27^, ^28^, ^29^ In the present study, MCP‐1 was obviously increased in the pancreas of CP mice (Figure 6C–F). Furthermore, the IHC results showed that PSC was one of the MCP‐1‐positively stained cells, besides atrophied acinar cells and inflammatory cells. In vitro, overexpression of MCP‐1 in PSCs stimulated with TGF‐β1 was detected both in the supernatant of the cells and cell lysate (Figure 6A,B). These results confirm that MCP‐1 could be produced by activating PSC. Moreover, we found that the production of MCP‐1 was significantly reduced by transfection of si‐p65 RNA in the PSCs (Figure 6A,B). These results imply that NF‐κB in PSCs may be considered a target for the regulation of MCP‐1 production.

However, the effects of a high level of MCP‐1 produced by PSCs on the progression of inflammation and fibrosis in CP are unclear. It is established that MCP‐1 recruits monocytes, T cells and dendritic cells to injured areas.^27^, ^28^, ^29^ According to our results demonstrating increased macrophage infiltration in the in vivo experiments (Figure 6G) and overexpression of MCP‐1 in PSCs in vitro (Figure 6E,F), we speculated that overexpression of MCP‐1 in PSCs may possibly influence macrophage infiltration.

We further isolated BMDMs and incubated them with supernatant containing a high concentration of MCP‐1. The scratch wound healing assay was performed to observe the migration of macrophages. The results reveal that the migration of BMDMs was markedly promoted after 24 hours of incubation with the supernatant of PSCs stimulated with TGF‐β1. However, the culture supernatant of PSCs with NF‐κBp65 siRNA significantly inhibited the migration of BMDMs (Figure 6H,I). These results further verified that the chemokine MCP‐1 secreted by PSCs can recruit more macrophages to the injured pancreas and aggravate the pancreatic inflammatory progress.^30^ In addition, macrophage migration induced by the supernatant of activated PSCs was dependent on the activity of the NF‐κB pathway. In summary, NF‐κB activation precipitated the production of MCP‐1, which possibly recruited tissue‐infiltrating macrophages to participate in the progression of pancreatic inflammatory damage.

In the present study, we demonstrated the mechanism of NF‐κB signalling pathway in PSCs influencing pancreatic inflammation and fibrosis of CP (Figure 7). This study revealed the cascade as follows: In pancreatic inflammation, overexpressed TGF‐β1 bound to the TGF‐β receptor 1 of PSCs and induced TAK1 phosphorylation, as well as activation of the NF‐κB signalling pathway. NF‐κB activation further promoted an imbalance of MMP‐1/TIMP‐1, which increased the production of ECM and aggravated pancreatic fibrosis. On the other hand, NF‐κB activation promoted the secretion of MCP‐1, which recruited more macrophages to the damaged area and accelerated pancreatic inflammation.

The current study was the first to investigate the specific role of NF‐κB in isolated and cultured PSCs stimulated by TGF‐β1. Using RNAi, we yielded reliable results showing that NF‐κB is essential in promoting the activation of PSCs and the secretion of MCP‐1 and fibrosis‐related factors. Even though our in vitro experiments provide solid results, showing the activation of NF‐kB pathway in isolated PSCs is an important event aggravating the progression of inflammation and fibrosis. The specific role of NF‐kB in PSCs should be depicted by the help of well‐established in vivo models, in hope of having a more concrete understanding about the role of NF‐κB in PSCs during CP and pancreatic fibrosis.

## CONFLICT OF INTEREST

The authors declare no conflict of interest.

## AUTHOR CONTRIBUTION

**Nan Wu:** Data curation (equal); Formal analysis (equal); Methodology (equal); Writing – original draft (equal); Writing – review and editing (equal). **Xiaofan Xu:** Data curation (equal); Formal analysis (equal); Methodology (equal); Writing – original draft (equal); Writing – review and editing (equal). **Jiaqi Xin:** Data curation (equal); Methodology (equal); Writing – review and editing (equal). **Jianwei Fan:** Data curation (equal); Methodology (equal); Writing – review and editing (equal). **Yuanyuan Wei:** Data curation (equal); Methodology (equal); Writing – review and editing (equal). **Qingxia Peng:** Data curation (equal); Methodology (equal); Writing – review and editing (equal). **Lifang Duan:** Data curation (equal); Methodology (equal); Writing – review and editing (equal). **Wei Wang:** Project administration (equal); Supervision (equal); Writing – original draft (equal); Writing – review and editing (equal). **Hong Zhang:** Conceptualization (lead); Funding acquisition (lead); Project administration (lead); Supervision (lead); Writing – original draft (lead); Writing – review and editing (lead).

## Supporting information

Fig S1

## Data Availability

The data that support the findings of this study are available from the corresponding author upon reasonable request.
