# Supplementary material for: The effects of nuclear factor‐kappa B in pancreatic stellate cells on inflammation and fibrosis of chronic pancreatitis
Source: J Cell Mol Med. 2020 Dec 30;25(4):2213–27. doi: 10.1111/jcmm.16213 (PMC7882951; doi:10.1111/jcmm.16213)
Supplement: Supplementary file 1 — Fig S1 [file JCMM-25-2213-s001.pdf]

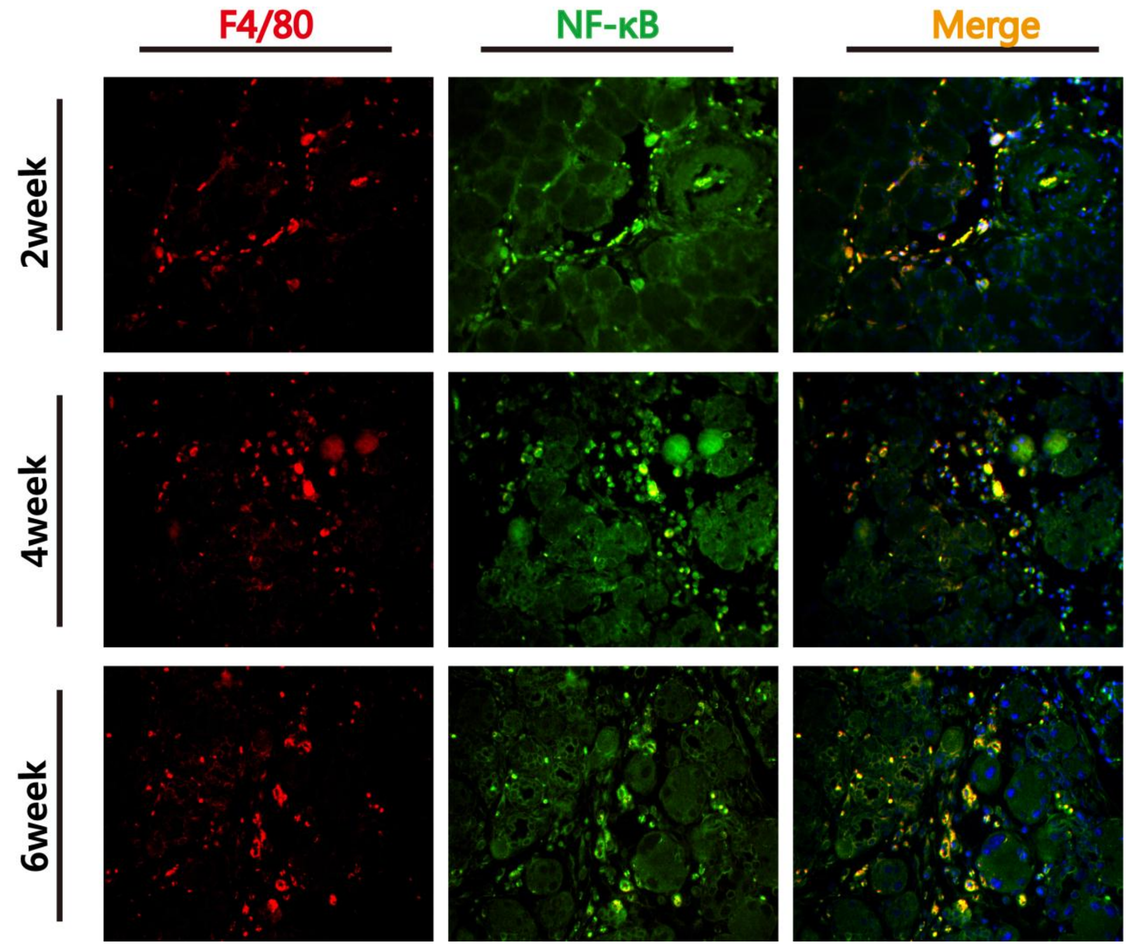

Figure S1. The co-staining of NF-kB and F4/80 (macrophages marker) in the murine models of CP. Dylight 594-conjugated F4/80 (red), Dylight 488-conjugated p65 (green), DAPI (blue), and co-expression areas (orange). Original magnification: 400×.
